# Identification of domains in *Plasmodium falciparum* proteins of unknown function using DALI search on AlphaFold predictions

**DOI:** 10.1038/s41598-024-60058-x

**Published:** 2024-05-08

**Authors:** Hannah Michaela Behrens, Tobias Spielmann

**Affiliations:** Bernhard Nocht Institute for Tropical Medicine, 20359 Hamburg, Germany

**Keywords:** Protein analysis, Protein function predictions

## Abstract

*Plasmodium falciparum*, the causative agent of malaria, poses a significant global health challenge, yet much of its biology remains elusive. A third of the genes in the *P. falciparum* genome lack annotations regarding their function, impeding our understanding of the parasite's biology. In this study, we employ structure predictions and the DALI search algorithm to analyse proteins encoded by uncharacterized genes in the reference strain 3D7 of *P. falciparum*. By comparing AlphaFold predictions to experimentally determined protein structures in the Protein Data Bank, we found similarities to known domains in 353 proteins of unknown function, shedding light on their potential functions. The lowest-scoring 5% of similarities were additionally validated using the size-independent TM-align algorithm, confirming the detected similarities in 88% of the cases. Notably, in over 70 *P. falciparum* proteins the presence of domains resembling heptatricopeptide repeats, which are typically involvement in RNA binding and processing, was detected. This suggests this family, which is important in transcription in mitochondria and apicoplasts, is much larger in *Plasmodium* parasites than previously thought. The results of this domain search provide a resource to the malaria research community that is expected to inform and enable experimental studies.

## Introduction

*Plasmodium falciparum*, the malaria parasite responsible for over 600 000 deaths every year^1^, has a complex life cycle and its biology is still only partly understood. Of its 5268 predicted genes, 1407 remain without any annotation (release 62, January 2023^2,3^). Annotations of genes are most often based on sequence similarity to well characterized proteins in other organisms^4^. As part of an experimental gene-by-gene analysis of unknown proteins encoded on *P. falciparum* chromosome 3 we found that structural similarities of AlphaFold structures matched well the experimental evidence of several proteins of unknown function^5^. This indicates that many parasite proteins have a conserved evolutionary origin but evolved beyond recognition on the primary sequence level but not on the structural level. It can therefore be assumed that comparisons of the structural prediction of unknown proteins will yield useful information on the functions of proteins currently annotated as unknown. The availability of AlphaFold structure predictions^6^ now enables the identification of domains based on geometric comparisons, independent of DNA and amino acid sequences. These similarities could provide information about gene products that so far lacked annotation. Here we apply this approach to all genes of unknown function in *P. falciparum*, specifically to the reference strain 3D7. Several sequence-independent algorithms exist to compare protein structures to each other based on geometry, including VAST^7^, DALI^8^, Foldseek^9^, CE^10^, the protein size-independent scoring algorithm TM-align^11^ and others (reviewed in^12^). For this screen we chose the DALI search algorithm as it has several advantages. It is set up to search the PDB and has an integrated graphical user (GUI) interface that also allows quick assessment of Pfam-annotated domains in the aligned structures. Further it has a built-in GUI for visual 3D-assessment of the alignment of hits to the query. We chose an open approach, where we screened all proteins of unknown function for the presence of any domains, rather than searching for a predefined set of domains or restricting the search to a small set of proteins of interest. This has the advantage that discoveries can be made independent of hypotheses that are related to specific processes in the parasite.

Using this approach, we here report the identification of domains with similarities to experimentally determined protein domains in 287 proteins encoded by genes of unknown function from *P. falciparum* 3D7 by an open-ended search. In addition, we used a targeted approach to search for proteins with similarity to the armadillo domain-containing ASA2 and ASA3 proteins which came to our attention as a frequent similarity in the open-ended approach. In this targeted search we found folds resembling heptatricopeptide repeats, which are typically involved in RNA-binding and -processing, in 53 previously un-annotated proteins, indicating that this family is much larger than previously though. We provide these results as a resource to the community to support experimental work.

## Results

### Validation of AlphaFold-DALI approach in *P. falciparum*

The aim of this work was to obtain information on *P. falciparum* proteins of unknown function by identifying structural similarities to known domains. For this we chose, what we refer to as the AlphaFold-DALI (AF-DALI) approach: the AlphaFold predictions of selected proteins were compared to experimentally determined structures in the protein data bank (PDB)^13^ using the search algorithm DALI^8^. Included in the search were proteins that contained "globular" folds, thus excluding structures which contained solely disordered, extended and fibrous folds, as this is a requirement for successful DALI searches. The search results from the DALI search were visually assessed to exclude structures that aligned only by two or fewer helices or beta strands. Similarities to annotated domains in the search results were considered relevant if they did not conflict with other domain annotations among the top hits.

**Figure 1:**
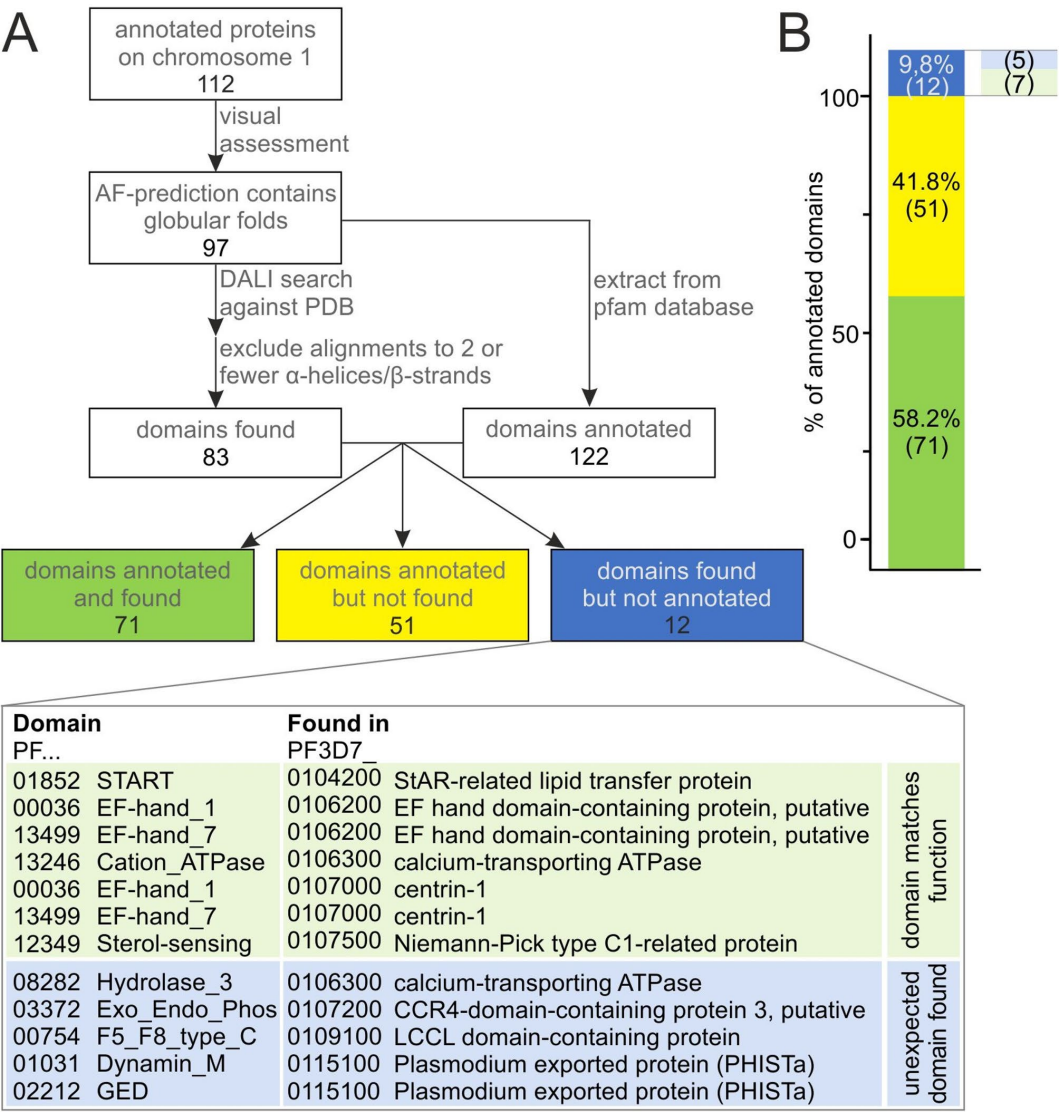
(**A**) Pipeline for detection of domains by the AlphaFold-DALI approach in annotated *P. falciparum* proteins on chromosome 1 and comparison with annotated domains. Domains that were detected by the AlphaFold-DALI approach but not annotated in the Pfam database (blue) were grouped according to their match with annotated functions (light green and light blue). (**B**) Proportions of detected and annotated domains on chromosome 1 from (**A**).

To assess whether this approach can reliably identify structural similarities of *P. falciparum* proteins to known domains, we first tested it on all annotated proteins encoded on chromosome 1 in *P. falciparum* 3D7. Out of the 112 proteins encoded on chromosome 1, the AlphaFold predictions of 97 contained globular folds and where thus suitable to be subjected to DALI searches. Out of 122 domains that where annotated in these proteins in the Pfam database (version 36), 71 (58%) were correctly detected by the AF-DALI approach (Fig. 1 green, Supplementary data 1). Of the domains that were not detected (Fig. 1 yellow), a quarter (14) were in three PfEMP1s, which have many domains. The AF-DALI approach favours the detection of the best domain rather than all domains, which can lead to a failure to detected domains, especially in proteins with many domains. In addition to the annotated domains, 12 domains were detected that were not annotated in the Pfam database (Fig. 1 blue) of which seven were in accordance with the protein’s function, for example the STARt domain which was previously detected in the START protein^14^ (Fig. 1 light green) and five were not expected to be found in the respective proteins but did not disagree with the annotated function either (Fig. 1 light blue, Supplementary data 1). Thus, the application of the DALI-search algorithm to AlphaFold-prediction structures of *P. falciparum* proteins reliably detects more than half of the annotated domains and rarely (5 of 134, Fig. 1 light blue) detects domains that might either be false positives or were previously unknown in these annotated proteins of chromosome 1. This indicated that this approach is not optimal to find new domains in already annotated *P. falciparum* proteins, likely because the DALI approach is not ideal to detect additional domains in these proteins and because annotation may already be comprehensive in some of these proteins. However, the detection rate of over 50% of known domains and the low false positive rate also indicated this AF-DALI approach is suitable to detect domains which might be indicative of function in the proteins of unknown function in *P. falciparum*.

### Identification of domains in proteins of unknown function by open-ended AF-DALI search

**Figure 2:**
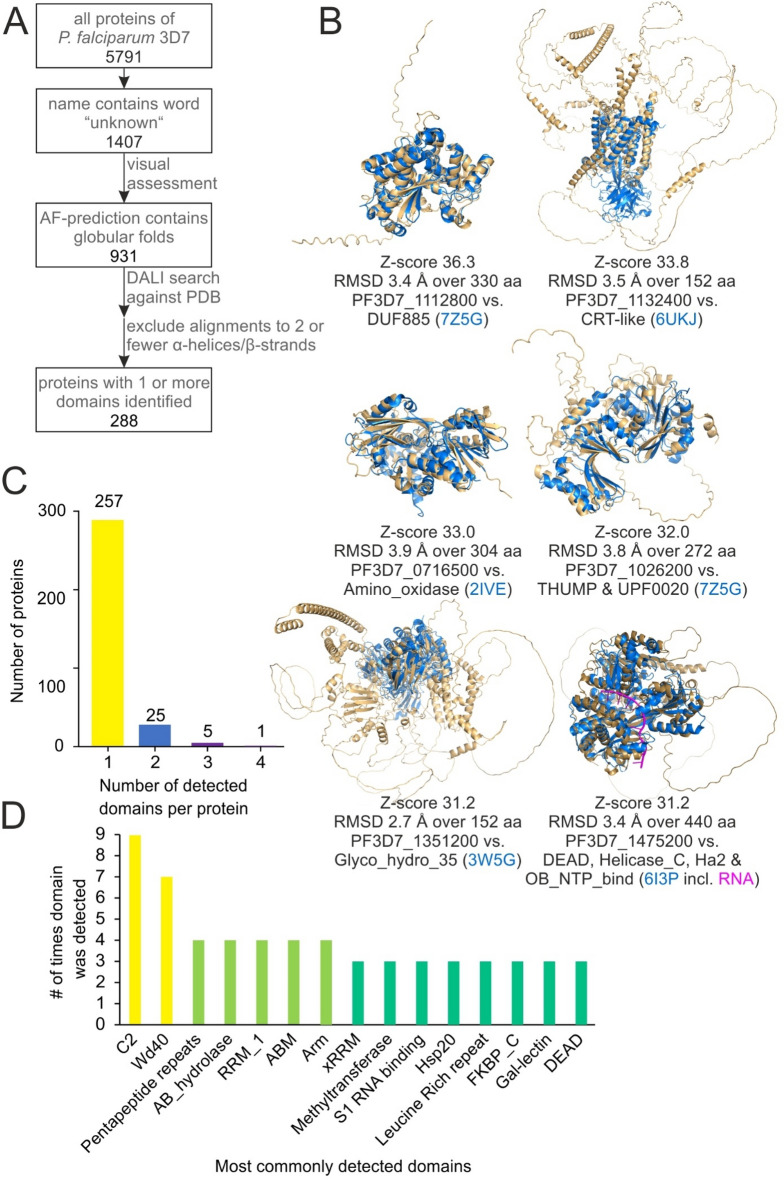
Identification of domains based on similarity of AlphaFold prediction to PDB structures. (**A**) AF-DALI approach pipeline for detection of domains in proteins of unknown function. (**B**) Alignment of AlphaFold prediction (beige) to experimentally determined structured (blue) with domain annotation, with Z-scores > 30. Indicated are the Z-score (defined as (S(A,B)-m(L))/(0.5m(L)), where S(A,B), is the DALI score, a weighed sum of C _*α*_–C _*α*_ distances between aligned residues of two proteins and m(L) is the expected score for a random pairwise comparison, depending on the average length of the two proteins L = (L_A_L_B_)^1/2^^16^) resulting from the DALI search, the root mean square deviation (RMSD) generated by Pymol cealign, the PlasmoDB gene accession number of the gene encoding the *P. falciparum* protein, the name(s) of the domain(s) in the experimentally determined structure according to Pfam and the PDB accession (blue) of the experimentally determined structure. (**C**) Number of proteins in which one, two, three or four domains were detected by the open-ended search. (**D**) Most commonly detected domains shown with number of times they were detected. Domain names as they appear in Pfam.

In order to identify known domains in *P. falciparum* proteins of unknown function, all 1407 proteins which were named protein of unknown function on PlasmoDB^15^ were selected for analysis. Included in the search were 931 proteins that contained globular folds. These were visually assessed to exclude structures that aligned only by two or fewer helices or beta strands, resulting in the identification of similarity to at least one annotated domain in 287 of the 1407 unknown proteins (Fig. 2A, Supplementary data 2).

**Figure 3:**
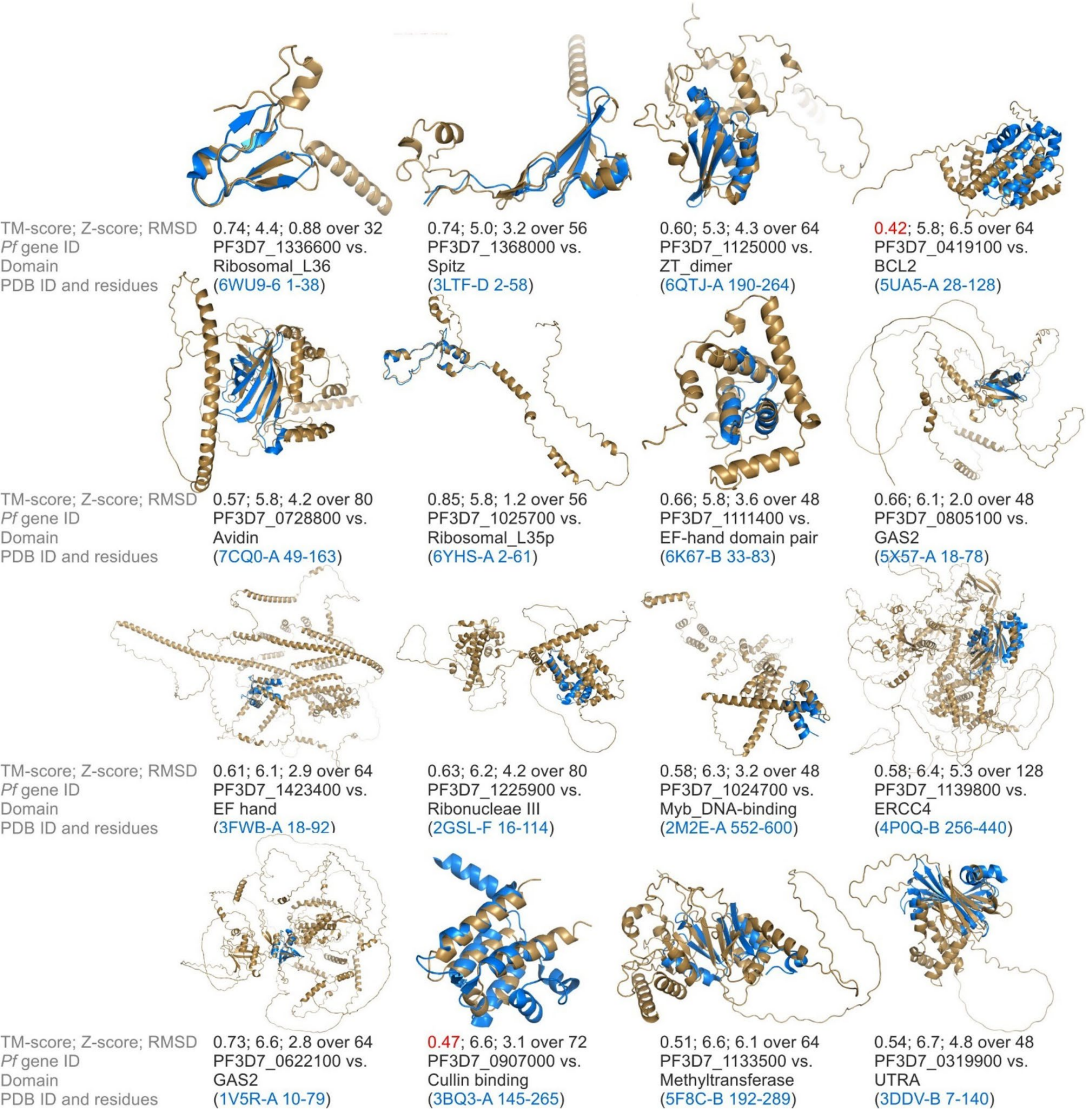
TM-align assessment of lowest-scoring 5% of aligned domains. The AlphaFold structure of the *P. falciparum* protein (beige) is aligned to the indicated residues of the PDB structure (blue), which corresponds to the annotated domain. Given are TM-score (defined as Max((1/L_Target_)Σ_i_^Lali^(1/1 + (d_i_/d_0_(L_Target_))^2^)) where L_Target_ is the length of the target protein, L_ali_ is the number of aligned residues, d_i_ the distance between the ith pair of aligned residues and d_0_ is a distance-normalization parameter^11^) generated by TM-align, Z-score generated by DALI, RMSD generated by Pymol cealign, PlasmoDB gene ID of *P. falciparum* gene that encodes the respective protein, name of the identified domain of similarity, the PDB ID of the structure that contained the domain and the amino acid residues at which the domain was found. For 3FWB-A residues 18 to 92 are shown even though the annotated domain only includes residues 24–52 because structural similarity extended to this region. TM-scores under 0.5 are shown in red.

The DALI search assigns a Z-score (a normalized weighted sum of similarities of distances between aligned residues^16^) to each alignment which reflects its quality, where Z-scores of below 2 indicate non-specific similarity^16^. While all except one of the 287 structure alignments that were considered specific and a good fit by the visual inspection had Z- scores of 5 or higher, PF3D7_1336600 had a lower Z-score (Supplementary data 2). However, the lower score in that case was due to its small size as it still showed a very good alignment (also shown in Fig. 3). The six highest-scoring similarities had Z-scores of over 30 (Fig. 2B). Of the proteins in which domains were identified, 254 contained one newly identified domain, 23 contained two and five proteins contained 3 newly identified domains (Fig. 2C, Supplementary data 2). As an example of a protein in which similarity to two domains was detected PF3D7_1013300 was visualized (Figure S1). Among the identified domains, most (> 200) domains occurred only once, 27 occurred twice and 17 occurred three times or more (Fig. 2D, Supplementary data 2).

To validate these results, the lowest-scoring 5% of similarities found (based on DALI Z-score), were analysed using a second algorithm. For this the algorithm TM-align was used which assesses geometric similarity of proteins independent of their size and reports the similarity as the TM-score^11^. This algorithm scores similarities between two protein structures on a scale from 0 to 1, where TM-scores over 0.5 indicate that searched structures contain the queried fold^11^. Of the 16 structures assessed 14 showed a TM-score over 0.5 (Fig. 3), when compared to the top annotated hit from the DALI search (which was an experimentally determined structure downloaded from the PDB and cropped to the domain that showed similarity). The remaining two showed TM-scores over 0.4. Thus, the two algorithms show good agreement even among the lowest-scored similarities, suggesting only a low proportion of false positive similarities. This was also expected based on our test population of annotated proteins on chromosome 1 which had less than 4% of possible false positives (Fig. 1).

### Comparison with published data

To assess whether the domain similarities we detected (Supplementary data 2) can provide information on protein function, we searched the literature for published experimental data on the proteins of unknown function. Although all of the 287 proteins for which similarity was found are encoded by genes that are annotated as of unknown function, some experimental data exists for nine of them, mainly localization data but also some data on protein interactions and essentiality (Table 1). The cellular location of the proteins encoded by PF3D7_0307600, PF3D7_0313400 and PF3D7_0319900 was previously studied by fusing the endogenous gene with the sequence encoding GFP in an experimental screen of genes of unknown function from chromosome three in *P. falciparum* 3D7 parasites^5^ (Table 1). PF3D7_0205600, PF3D7_1013300 and PF3D7_1329500 were analysed by the same approach^17^^18,19^ while the *P. berghei* ortholog of PF3D7_1132400 was expressed as an endogenous HA-fusion^20^ and their cellular locations observed (Table 1). The *P. berghei homolog* of PF3D7_1420500 was recently identified as a component of the ARP2/3 complex^21^ and for PF3D7_0903600 functional data of the orthologue in *Toxoplasma gondii* recently became available^22^. Using this published information, we next assessed whether the AF-DALI-detected domains were plausible. PF3D7_0205600 and PF3D7_1329500 were located in the nucleus^17,19^, supporting that the identified regions with similarity to CDC45-like and TIG domains, which are found in DNA replication proteins and transcription factors, respectively, might serve similar functions.

**Table 1:** Comparison of published data with predicted domains.

PlasmoDB ID	Newly identified domain and its function	Experimental data available for protein	Match of domain identification and experimental data
PF3D7_0205600	CDC45-like; DNA replication (Z-score 28.0)	GFP-fusion localizes to nucleus and cytoplasm^18^	Predicted function supported by experimental data
PF3D7_0307600	Rad51; DNA repair (Z-score 24.2)	GFP-fusion gives unknown localization pattern within the parasite^5^	Predicted function not supported by experimental data Expected would be nuclear localization
PF3D7_0313400	AAA; chaperone (Z-score 13.5)	GFP-fusion localizes to the parasite cytoplasm^5^	Predicted function possible based on experimental data
PF3D7_0319900	UTRA; Ligand-binding, modulates transcription factor activity in response to small molecules (Z-score 6.7)	GFP-fusion gives unknown localization pattern in the parasite^5^	Predicted function possible based on experimental data
PF3D7_0903600	HOOK_N; dynein-associated cargo adaptor proteins (Z-score 12.0)	*Toxoplasma gondii* homolog *Tg*HOOK regulates apical positioning and secretion of micronemes and contributes to egress, motility, host cell attachment, and invasion. Interacts with homologs of HOOK interaction partners TgFTS or TgHIP.^22^	Predicted function supported by experimental data
PF3D7_1013300	Ncstrn_small (small lobe) and Nicastrin (large lobe); Two domains making up nicastrin, a part of complex for intra-membrane proteolysis of integral membrane proteins. This protein is modified in Golgi or trans-Golgi network (Z-score 20.4)	GFP-fusion localizes to the periphery of ring stages, trophozoites and schizonts and is visible in ER in trophozoites and schizonts^19^ Isolated from detergent-resistant membranes. HA-fusion gives “dotted labelling within the parasite cytoplasm “^27^	Predicted function supported by experimental data
PF3D7_1132400	CRT-like; chloroquine resistance transporter and homologues. Arabidopsis homologues involved in thiol transport from plastid to cytosol (Z-score 33.8)	In *P. berghei* non-nuclear, non-apicoplast signal of HA-fusion protein. Predicted to have 9 to 10 TM domains. Western blot shows electrophoresis anomalies common to membrane proteins^20^	Predicted function possible based on experimental data
PF3D7_1329500	TIG; in cell surface receptors and in transcription factors for DNA binding (Z-score 8.1)	GFP-fusion localizes to nucleus in ring and trophozoite stage, in addition foci in the parasite periphery at K13 compartment in trophozoites stage^17^	Predicted function supported by experimental data
PF3D7_1430500	P34-Arc; Component of Arp2/3 complex, which controls actin polymerization (Z-score 18.1)	Coimmunoprecipiates and coexpressed with ARPC1 and other ARP2/3 complex components in activated gametocytes^21^	Predicted function supported by experimental data

PF3D7_1013300 showed similarity to both domains that make up nicastrins^23^and equally has a transmembrane domain and a short C-terminal tail (Figure S1). In other organisms nicastrin is part of the gamma secretase protein complex that proteolytically processes integral membrane proteins^24^. Because inhibitors against the human gamma secretase complex had no effect on *P. falciparum*^25^ and homologs of the complex components were not found by sequence similarity, the gamma secretase complex was thought to be absent in *Plasmodium* parasites. Yet, the location of PF3D7_1013300 in the ER and the cell periphery would be consistent with nicastrin in other systems where it gets modified in the Golgi before transport to its final destination at the plasma membrane. Thus, the experimental data is compatible with the observed structural similarity and suggests that at least the nicastrin component of the gamma secretase complex is present in *P. falciparum* parasites.

We found that PF3D7_1430500 is similar to the P34-Arc domain (PF04045), which is part of the Arp2/3 complex that canonically nucleates actin filaments. The *P. berghei* homolog of PF3D7_1430500, now called PbARPC2, was found to be a homolog of the human P34-Arc protein hARPC2^21^. PbARPC2 coimmunoprecipitated with and was coexpressed with other proteins that bear structural similarity to subunits of the Arp2/3 complex. It was confirmed that this complex is functional and takes an essential role in the DNA segregation and maturation of *P. berghei* male gametocytes. Overall this suggests that PF3D7_1403500 is a P34-Arc homolog as suggested by our AF-DALI search.

For PF3D7_0903600 a HOOK_N domain-like fold was detected, which typically occurs in dynein-associated cargo adaptors. It’s ortholog in *Toxoplasma gondii* has recently been shown to interact with typical hook-interacting proteins and be involved in processes typical for hook proteins^22^, giving credibility to the DALI-search detected domain.

The domains identified in PF3D7_0313400 (AAA domain), PF3D7_0319900 (UTRA domain) and PF3D7_1132400 (CRT-like domain) can occur in proteins of various cellular locations and thus the experimentally observed cellular locations neither disagree with nor confirm their potential functions. As such, the domain predictions are possible based on the available experimental data. Solely for PF3D7_0307600, which we found to harbour similarity to a Rad51 domain (typically involved in DNA repair), the GFP-fusion protein was—contrary to our expectation—not found in the nucleus^5^. This might be due to a false AF-DALI search result, a repurposing of the domain in this protein or an altered cellular location due to the GFP-fusion, which is possible given it is not essential^5,26^.

### Proteins containing Armadillo-like domains

Coincidentally, we noticed during the DALI-search that many unknown *P. falciparum* proteins showed structural similarity to “Mitochondrial ATP synthase subunit” (ASA2) and “Mitochondrial F1F0 ATP synthase associated 32 kDa protein” (ASA3). These two proteins are part of the Polytomella F-ATP synthase complex for which structures of several states were determined by single-particle cryo-electron microscopy^28^. No domains were annotated in the ASA2 and ASA3 proteins according to the Interpro database^29^, which integrates domain annotations from its member databases including the Pfam database which is used by DALI. As a result, using our approach we could not assign domains in the *P. falciparum* proteins that were structurally similar to ASA2 and ASA3. The fact that similarity to domains cannot be detected when said domains are not annotated in the proteins with experimental structures, is a systematic limitation of the AF-DALI approach. To exemplarily show that detecting even more domain similarities is possible when new domains are annotated in the PDB structures, we decided to manually determine which domains are present in ASA2 and ASA3 by DALI search and to use this to search for similar domains among the *P. falciparum* proteins of unknown function.

To manually determine the domains in ASA2 and ASA3 we performed DALI searches with these proteins against all PDB structures, using their structures from the PDB (PDB 6rd4). The top hits for either protein were ASA2 and ASA3, suggesting that both were very similar to each other. Further both were similar to proteins containing armadillo domains (PF00514), for example in catenin delta (PDB 3L6X^30^), plakoglobin (PDB 3IFQ^31^), plakophilin (PDB 1XM9^32^) and several other proteins, suggesting that both ASA2 and ASA3 contain domains, which are made up of several armadillo repeats. We thus concluded that ASA2 and ASA3 contain a type of armadillo domain.

**Figure 4:**
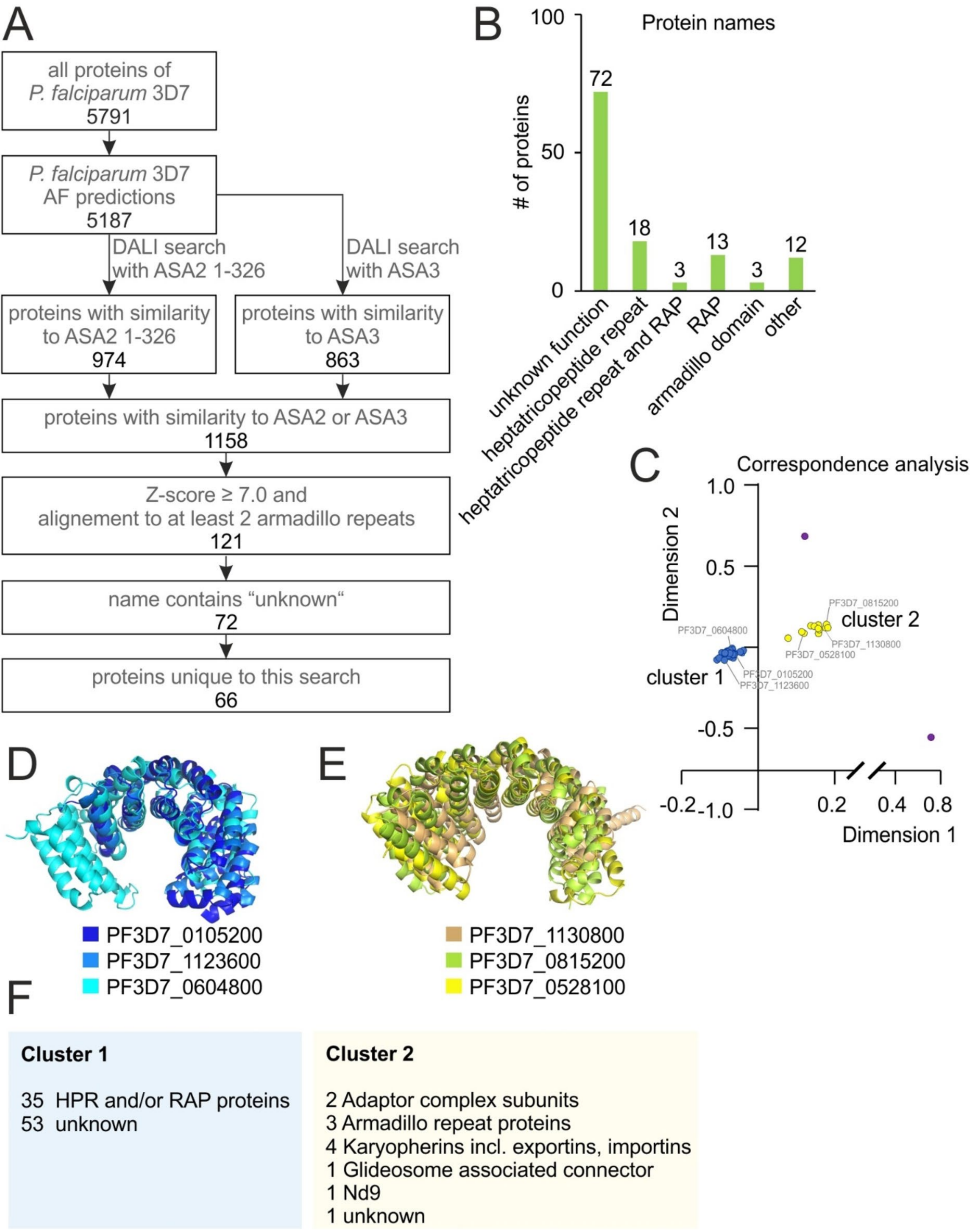
Identification of ASA2/3-like armadillo domains in *P. falciparum* proteins using AlphaFold predictions. (**A**) Pipeline for detection of ASA2/3-like armadillo domains. (**B**) Annotations of *P. falciparum* proteins which were found to be similar to ASA2 and ASA3. (**C**) Correspondence analysis to cluster annotated *P. falciparum* proteins with similarity to ASA2 and ASA3 by structural similarity to each other. Dots corresponding to proteins shown in (**D**) and (**E**) are labelled. Purple dots do not belong to cluster 1 or 2. (**D**) Three representative structures selected from cluster 1 as defined in (**C**) aligned to each other using cealign in Pymol. (**E**) Three representative structures selected from cluster 2 as defined in (**C**). Structures shown in (**D**) and (**E**) were used to sort *P. falciparum* proteins of unknown function with similarity to ASA2 and ASA3 into clusters 1 and 2. (**F**) Number of proteins of different functions in cluster 1 and 2.

We then performed DALI searches of these armadillo domains from ASA2 (residues 1-326) and ASA3 (full length) against all AlphaFold-predicted structures of *P. falciparum* in the database. 1158 genes were found to encode proteins that contains domains similar to ASA2, ASA3 or both. As the Z-score is dependent on protein size, a suitable cut-off had to be determined for the common domain of ASA2 and ASA3. Spot checks of hits across different Z-scores were performed and showed that hits with a Z-score of 6.5 or higher showed good similarity to ASA2 or ASA3, with alignment of more than three armadillo repeats which each consist of one helix pair. To avoid false positives, an even more stringent Z-score cut-off of 7.0 was chosen which resulted in 121 *P. falciparum* (in 3D7) protein hits (Fig. 4A, Supplementary data 3). All of them were found in both searches (ASA2 and ASA3). The hits included 18 heptatricopeptide repeat proteins, 13 RAP (**R**NA-binding domain abundant in **Api**complexans) proteins—both families are thought to be RNA-binding^33,34^,—3 that contain heptatricopeptide repeats and a RAP domain, 3 proteins that are named armadillo-domain containing proteins, 72 proteins of unknown function and 12 proteins with other annotations (Fig. 4B). In 6 of these, there were also new domains identified in the open-ended DALI search shown in Fig. 2, including DUF559, Importin-beta, Exportin 1-like, Armadillo domain and Atypical Armadillo domain. Both DUF559 domains (in PF3D7_0104600 and PF3D7_1207900) did not overlap with the identified armadillo domain, while the remaining four covered the same residues for which similarity to the ASA2-ASA3-armadillo domain was found (Figure S2).

The amino acid sequences of these 121 proteins were aligned using Clustal Omega^35^. The sequences of the proteins with annotation did not cluster according to their PlasmoDB annotations and generally showed little amino acid sequence similarity. We therefore decided to cluster them by structure. However, the 121 *P. falciparum* hits with similarity to ASA2 and ASA3 were too many to do this in one batch, thus first the AlphaFold predictions of only those with an annotation in PlasmoDB were clustered using the DALI all-against-all algorithm. Two distinct clusters were observed and named cluster 1 and cluster 2 (Fig. 4C). Cluster 1 was found to contain all heptatricopeptide repeat proteins, all RAP proteins and ARM2 (Fig. 4C–F). All of these proteins are known or predicted to be RNA-binding proteins^33,34,36^, while all proteins in cluster 2 are protein-binding proteins, some of which can also bind RNA^37–44^. Three structures were randomly chosen to represent each cluster (Fig. 4C–E) and aligned to batches of the 72 unknown *P. falciparum* proteins that harboured similarity to ASA2 and ASA3 (Figure S3). This assigned 53 new proteins to cluster 1 and one new protein to cluster 2, while 18 proteins could not be assigned to either cluster (Fig. 4F).

Of the 20 proteins previously known to contain RAP domains, 5 were known to also contain heptatricopeptide repeats. Of the remaining 15 RAP proteins, 13 showed up in our search, suggesting that these also contain heptatricopeptide repeats. The DUF559 domains identified in two newly identified heptatricopeptide repeat-like proteins also show some similarity to RAP domains. Thus, the co-occurrence of RAP domains with heptatricopeptide repeats in the same protein seems to be a common combination.

## Discussion

In this study we used the DALI algorithm to search for similarities of the available AlphaFold-predicted structures of all unknown *P. falciparum* (3D7) proteins to known domains in experimentally determined structures. This similarity search reduced the number of proteins without any annotation from 1407 to 1054, which is a 25% reduction. We expect the resulting set of several hundred identified domains (Supplementary data 2 and 3) to be a useful resource to the malaria research community that will help streamline further annotation and characterization efforts, and expected it to aid the understanding of the molecular mechanisms in malaria parasites. It provides information on the potential function of these *P. falciparum* protein inferred from the structural similarity to known proteins in other organisms. Additionally, in combination with functional and interactome data these domain searches can aid and substantiate the assignment of proteins to protein groups or complexes which together serve a specific function as e.g. recently done for proteins in mitosis^5,45^.

Open-ended approaches have the advantage that they can discover unexpected components and pathways. The search in this study gives hints to the unexpected presence of nicastrin, which is part of a functionality thought to be absent in *Plasmodium* parasites. It also identified a component of the ARP2/3 complex (PF3D7_1430500, Supplementary data 2) which was believed to be absent in the parasite but recently was identified^21^. Further, this approach can provide hints for proteins that were expected to be present but had so far not been identified. One example from our dataset is PF3D7_0404300 which shows similarity to Ran-binding proteins and could be a missing component in transport in and out of the nucleus.

Unexpectedly, we discovered a large group of proteins in the parasite with very similar domains, including all known heptatricopeptide repeat and RAP proteins as well as 53 unknown proteins (Supplementary data 3). Proteins containing heptatricopeptide repeats are known to bind and process polycistronic precursor RNAs into mRNAs and rRNAs for the mitochondrial ribosomes and to stabilize mRNAs to prevent decay^33^. Heptatricopeptide repeats proteins contain 37 amino acid long repeats, while proteins from the related families of pentatricopepide repeat proteins and octatricopeptide repeats proteins contain repeats which are 35 and 38 amino acids in length. Hepta-, penta- and octatricopeptide repeat proteins perform comparable RNA binding and processing functions, yet their distribution among different organism groups, like plants, apicomplexans and animals, varies^33^. In humans only 6 heptatricopeptide repeat proteins have been detected while around 70 were predicted in plants and dinoflagellates. In plants this is in addition to ~ 450 pentatricopepide repeat proteins^46^. In addition to the 18 previously described heptatricopeptide repeat proteins in *Plasmodium*, we here identified many more potential members of this group. If all 54 unknown proteins in cluster 1 of our clustering of the ASA2 and ASA3 hits indeed are such RNA-binding proteins, this would place the number of heptatricopeptide repeat-like RNA-binding proteins in *Plasmodium* (72) in a similar range as in plants. Interestingly, while some the *P. falciparum* proteins with similarity to heptatricopeptide repeat proteins contain insertions that break the 37-amino acid repeat motif that gives this domain its name, the geometry of the 37-amino acid repeat seems conserved.

Another interesting aspect is that we found heptatricopeptide repeat-like folds in a large proportion of proteins containing RAP and RAP-like DUF559 domains. RAP domains consist of a restriction endonuclease-like fold and are found in RNA-binding proteins^34^. Thus, heptatricopeptide repeats and RAP domains seem to be a common combination, suggesting that the two domains might act in concert.

While we believe this search to provide a valuable and useful resource, there are a number of limitations. The first inherent limitation is that this study used predicted structures of *P. falciparum* proteins rather than experimentally determined structures, which should not be viewed with the same confidence as experimentally determined structures. This circumstance has been discussed extensively following the introduction of AlphaFold^47–49^.

A second limitation was that the search presented here was not exhaustive. To the predicted structures, we applied DALI, the only search platform with which it was feasible to manually analyse several hundred protein structures in a reasonable time. This throughput is largely possible because of the built-in alignment and domain annotation viewers. The DALI algorithm scores search results based on length and quality of the structure alignment of the whole query structure. As a result, this algorithm favoured the discovery of one domain of highest confidence, while further domains in the same protein were only discovered if they incidentally had similar scores as the first domain or if they occurred in the same experimentally determined structure that the query aligned to. This is one reason, why the search presented in this study was not exhaustive, as also seen for the set of annotated proteins on chromosome 1 which were analysed as a control group, where 57.7% of the annotated domains were found (Fig. 1, Supplementary data 1). Other search algorithms could circumvent this drawback, for example the VAST algorithm first detects potential domains in the query structure and then performs searches for each of them individually in addition to the search with the complete query structure. Generally, the use of other search algorithms, such as VAST^7^, Foldseek^9^ and PDBeFold^50^, might result in the discovery of different domain similarities due to their different implementations and ways of scoring similarity. This search being not exhaustive is an important limitation that should be kept in mind when using these findings. Nevertheless, some of the identified domains already give clear functional indications and a protein of interest can then be analysed in more detail using other algorithms in which case further domains might be identified.

A third limitation of our approach is that domains that were not annotated in experimentally determined structures are not found. The search with ASA2 and ASA3 shows, based on a single example, how a missing domain annotation in experimentally determined structures can limit the search outcome of the AF-DALI approach. Manually providing this annotation showed that already for a single domain type this had a big impact. An even bigger increase in detected domains can be expected when several new domains are added to the Pfam database itself, as can be expected with updates of the database. It might therefore be fruitful to repeat the open-ended search as presented in Fig. 2 after future updates to the Pfam database. Another option to overcome this limitation and expand the number of detected domains would be to search against predicted rather than experimentally determined structures of well-studied and annotated proteins. Of course, with the caveat that predicted structures harbour some uncertainty.

Finally, the identified domains were not all computationally and none were experimentally validated in this study. Computational validation could be achieved by re-assessing the detected similarities using a second algorithm. This could for instance be the cealign command in Pymol (Fig. 2B) followed by confirming or rejecting annotations based on a cut-off score, as we have done previously^17^. It could also be done with the TM-align algorithm which has a pre-defined cut-off^11^ and was here used to validate the lowest 5% of the hits from this work. This confirmed 14 of 16 hits, overall giving a good credibility for the detected similarities, particularly as it can be assumed that the hits with higher scores are more reliable. Experimental validation is the gold standard for any structure prediction^51^. The assessment of available data in published literature on nine proteins annotated here, suggests that the majority of similarities found might align with the function that can be inferred from the domain similarities. However, a case-by-case validation is the only way to know whether the domains were identified correctly and whether these domains serve the same function in *P. falciparum* as in other organisms. We did not embark on a systematic experimental validation as this would take a considerable amount of time and we believe that rapidly providing these search results to the wider community will be more beneficial.

In conclusion, the results presented here are an example for the power of sequence-independent structure comparison approaches for reducing the number of genes lacking annotation and information on their potential biological function. Here applied to *P. falciparum*, it sheds light on the biology of the malaria parasite and provides starting points for future research which will lead to a better understanding of the biology of this pathogen.

## Methods

### Open-ended AF-DALI search

Lists of genes were downloaded from PlasmoDB v61 based on genomic location. For the search concerning proteins of unknown function, all genes which contained the word “unknown” in the gene name, for example “conserved Plasmodium protein, unknown function”, were included in the analysis. For the control group all genes which contained the word “unknown” in the gene name were excluded. Pseudogenes were excluded.

Available AlphaFold (alphafold.ebi.ac.uk, accessed December 2022-April 2024)^6^ structures were visually assessed for a globular and compact fold which is a prerequisite for a successful DALI search using the integrated 3D-viewer on alphafold.ebi.ac.uk^8^. The suitable AlphaFold structures were submitted to a DALI PDB search as available in the web application (http://ekhidna2.biocenter.helsinki.fi/dali/, accessed December 2022-Mai 2023). For this, the “PDB” tab was selected, the .pdb file of each AlphaFold structure was uploaded using the “choose file” option and the search started using the “submit” option. The search is performed against a mirror image of the PDB database from RCSB (www.rcsb.org), which is updated weekly. To analyse the results, the “matches against PDB90” were opened. The hits with the highest Z-scores were visually assessed for suitable alignment (excluded alignment of just 2 or fewer α-helices or β-strands) in the DALI 3D visualization tool. Of the top seven to ten hits with good alignment, the Pfam 35.0^52^ domain annotations were viewed in the DALI PFAM tool. A domain annotation for a region of a PDB hit that aligned to the query AlphaFold structure was considered a reliable hit if there were no conflicting domain annotation. Domain annotations were considered conflicting if other proteins with differently annotated domains aligned to the same amino acid residues and had a Z-score within 1.0 of the protein harbouring the domain of interest.

### Validation with TM-align

The 5% of domains with the lowest Z-score resulting from the open-ended DALI search were further validated. For each of these, the PDB structure of the top hit of the DALI search was cropped to only contain the residues annotated as the domain for which similarity was found. Where the top hit did not contain this annotation, the highest-scoring PDB structure with this annotation was used. The AlphaFold structure was compared with this cropped PDB structure using TM-align^11^ as available in the web application (https://seq2fun.dcmb.med.umich.edu//TM-align/, accessed December 2022-Mai 2023). The TM-score based on the length of the cropped PDB structure was assessed.

### ASA2/ASA3 search

All protein structures predicted for *P. falciparum* by AlphaFold^6^ were searched using ASA2 residues 1-326 and full length ASA3 (chain 2 and 3 of PDB 6rd4^28^) using the DALI AF search^8^. Alignments were visually assessed for at least 3 aligned armadillo repeats (helix pairs), resulting in all proteins with a Z-score larger or equal to 7.0 to be included for further analysis.

### Visualization and further assessment

Protein structures were analysed and visualized using PyMol 2.4.0 (Schrödinger, USA). Alignments were performed using the PyMol command cealign. Figures were arranged in CorelDraw X6-8.

### Supplementary Information

Supplementary Information 1.

Supplementary Information 2.

Supplementary Information 3.

Supplementary Information 4.

## Data Availability

All data is available from the manuscript, the supplement or from the authors (HMB, hannah.behrens@bnitm.de and TS, spielmann@bnitm.de) upon request.
