# Supplementary material for: Identification of domains in Plasmodium falciparum proteins of unknown function using DALI search on AlphaFold predictions
Source: Sci Rep. 2024 May 8;14:10527. doi: 10.1038/s41598-024-60058-x (PMC11079077; doi:10.1038/s41598-024-60058-x)
Supplement: Supplementary file 4 — Supplementary Information 4. [file 41598_2024_60058_MOESM4_ESM.docx]

Supplementary Materials for

**Identification of domains in *Plasmodium falciparum* proteins of unknown function using DALI search on Alphafold predictions**

Hannah Michaela Behrens*, Tobias Spielmann^#^

* Corresponding author: hannah.behrens@bnitm.de

## ^#^ Corresponding author: spielmann@bnitm.de

**This PDF file includes:**

Figures S1 to S3

**In addition to this PDF file the supplement includes:**

Supplementary data file 1 – Open-ended domain search

Supplementary data file 2 – Armadillo ASA2 ASA3 searches

Supplementary Figure 1
**
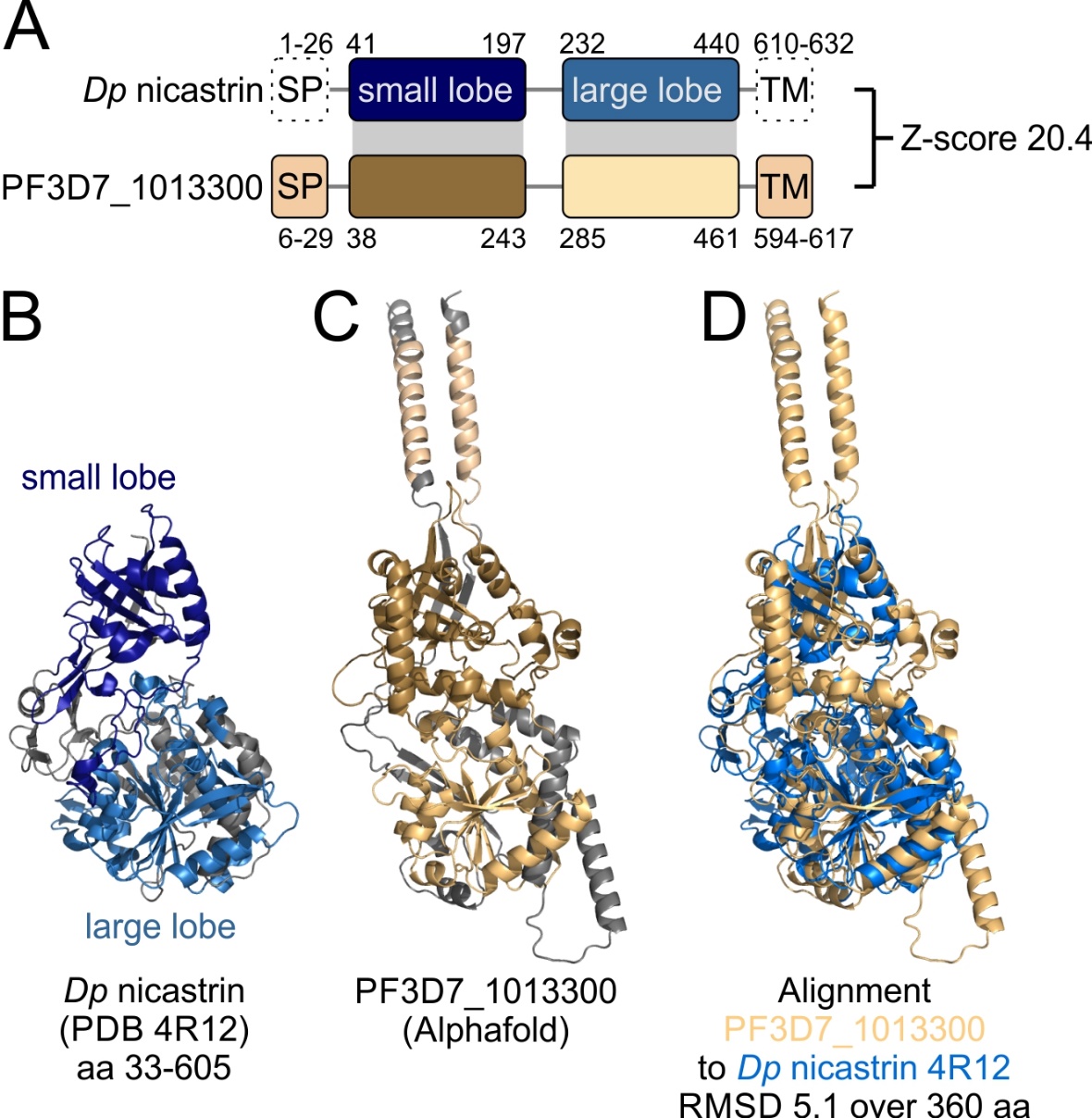
**

**Supplementary Figure 1: Nicastrin domains detected in PF3D7_1013300.** (A) Schematic representation of *Dictyostelium purpureum* nicastrin and PF3D7_1013300 (not to scale). Grey elements show corresponding domains. Z-score resulting from DALI score is indicated. Secondary structure elements of *D. purpureum* nicastrin that are not present in the crystal structure PDB 4R12 are shown with dashed lines. SP, predicted signal peptide; TM, transmembrane domain. (B) Crystal structure of *D. purpureum* nicastrin (PDB 4R12). Residues coloured as in (A). (C) Alphfold structure prediction of PF3D7_1013300. Residues coloured as in (A). (D) *D. purpureum* nicastrin (PDB 4R12, blue) and Alphfold structure prediction of PF3D7_1013300 (beige) aligned to each other using cealign in Pymol. RMSD generated by Pymol cealign is indicated.

Supplementary Figure 2

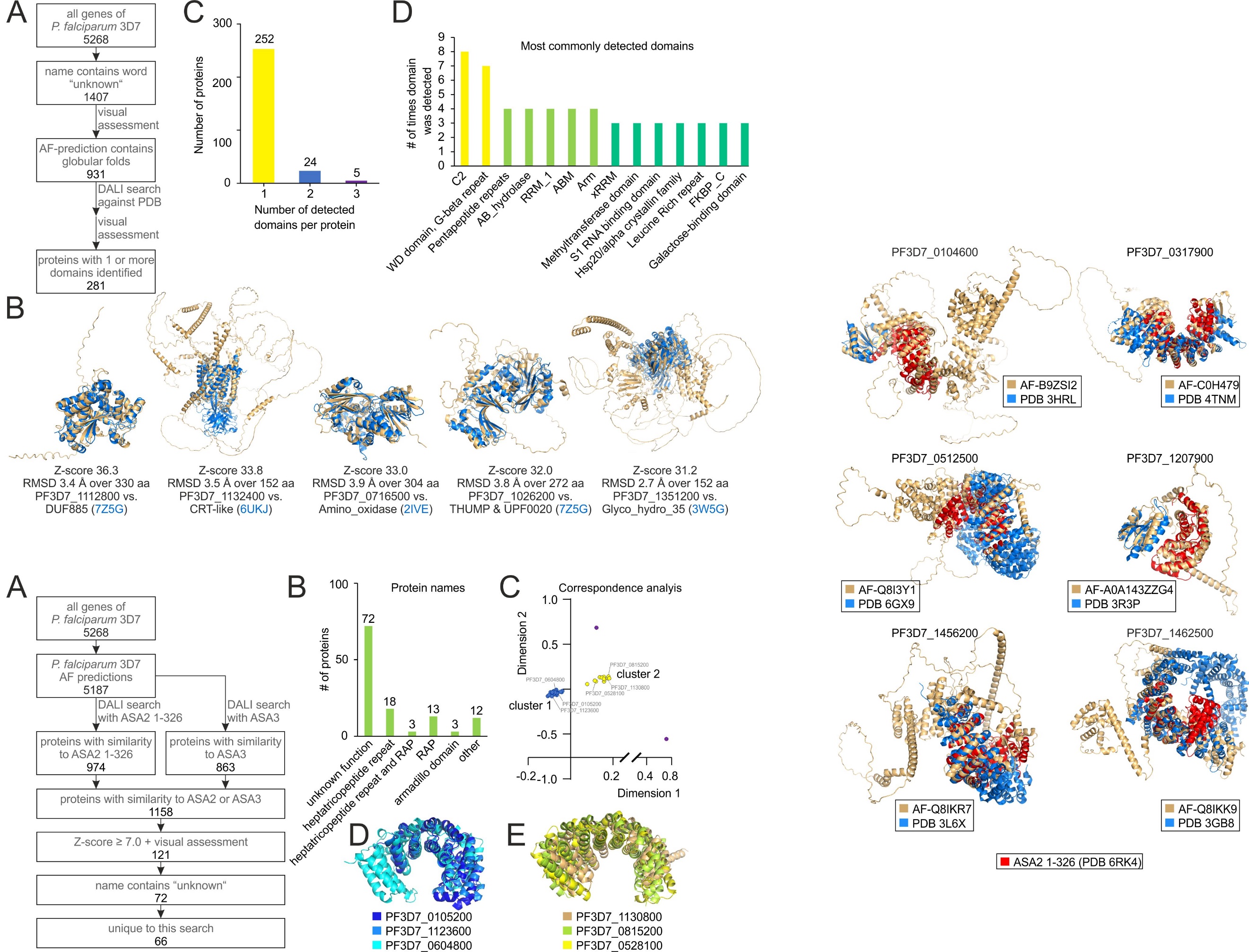


**Supplementary Figure 2: Spatial overlap of ASA2 with hits identified in open-ended domain search.** Alphafold structure predictions of proteins for which domains were found in the open-ended search and in the ASA2/ASA3-based search are shown (beige), and aligned to ASA2 residues 1-326 from PDB 6RK4 (red) and to the highest-scoring domain-annotated hit of the open-ended search (blue). For PF3D7_1207900 only ASA2 residues 1-170 are shown for clarity. For PF3D7_0104600 and PF3D7_1207900 the domains from the open-ended search (both DUF559, blue) and the armadillo domain (red) align to different regions of the target protein. For the other four structures the domains align to the same region of the target protein.

Supplementary Figure 3

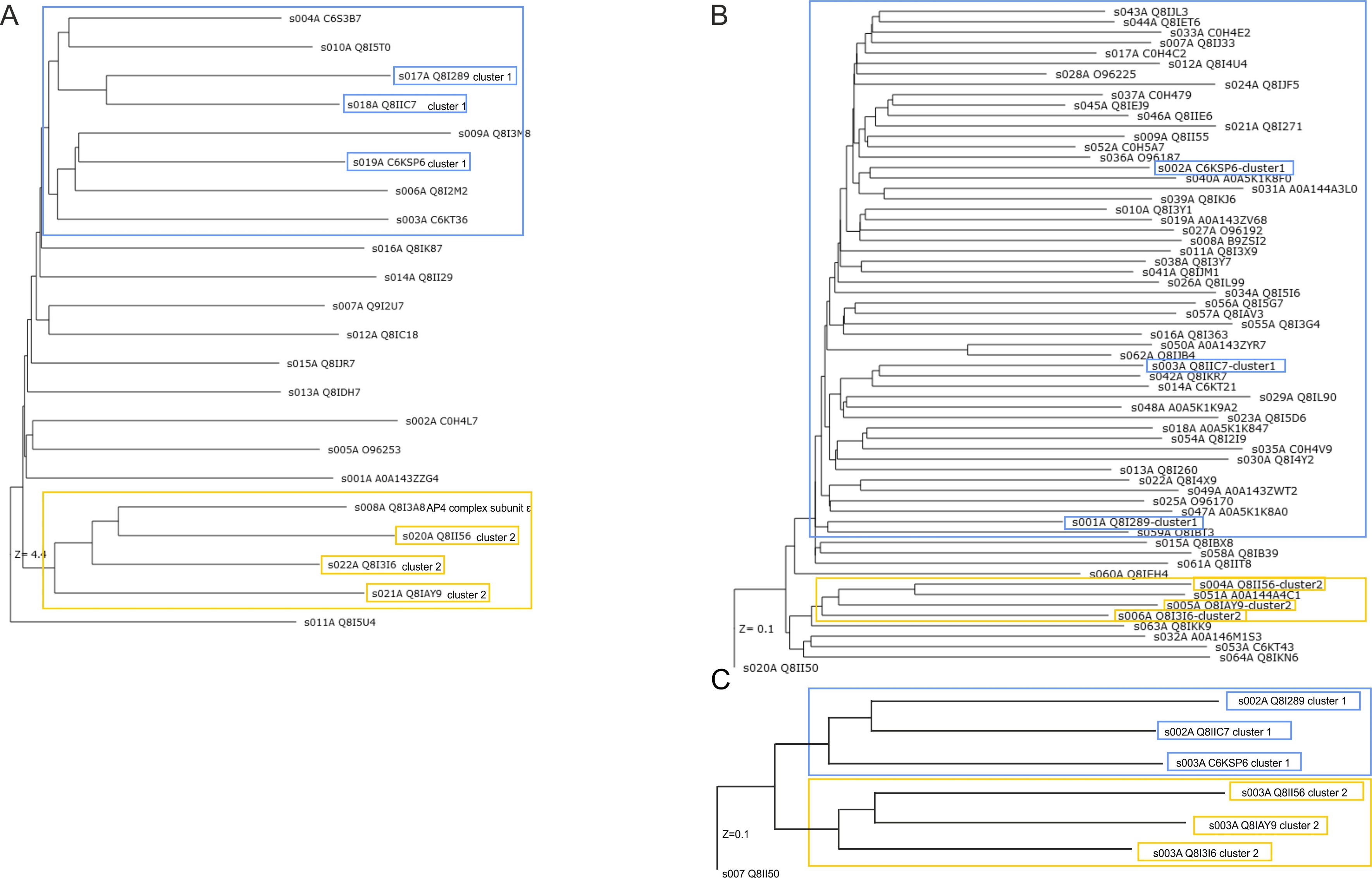


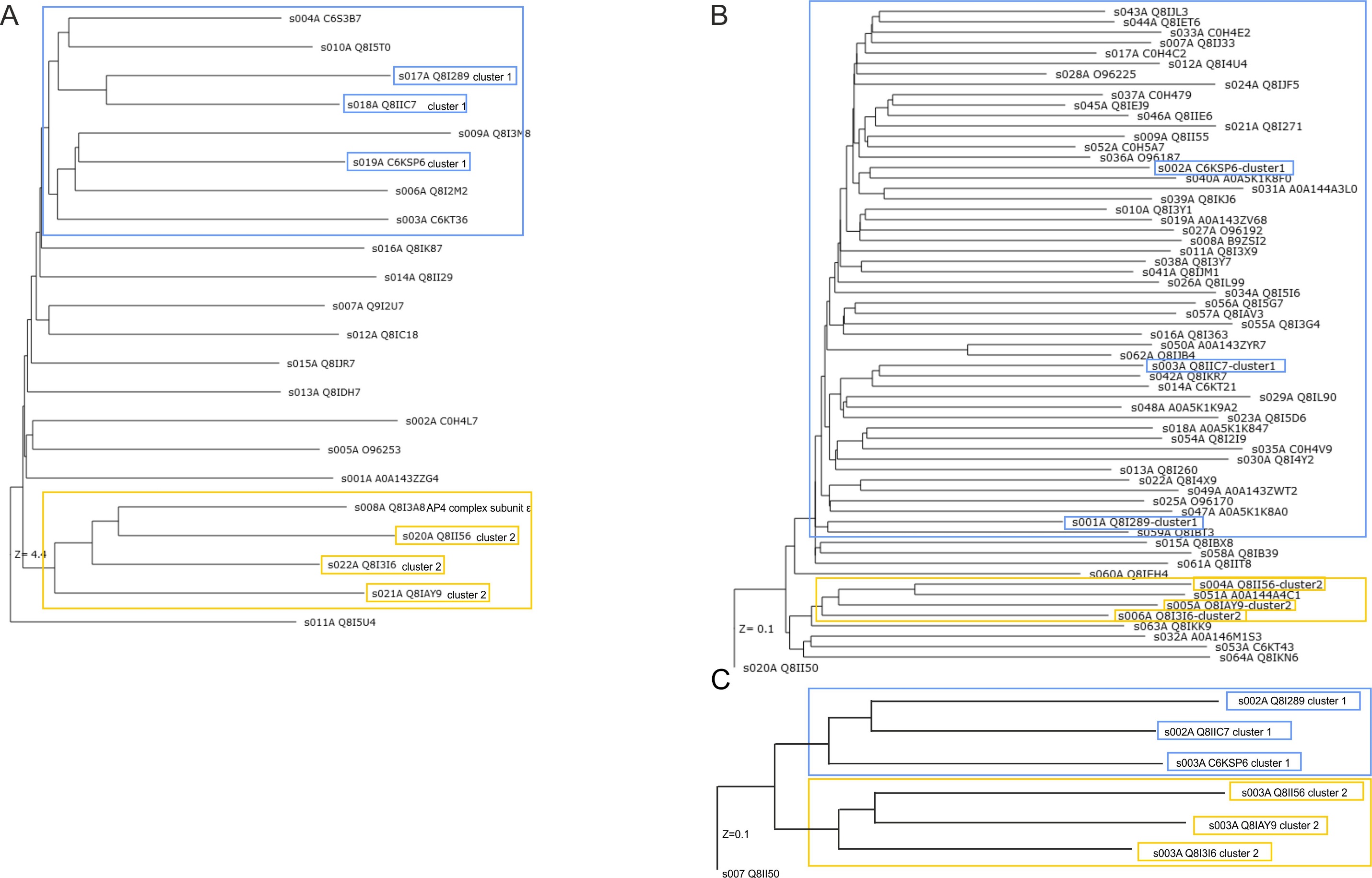


**Supplementary Figure 3: Structure based clustering of unknown proteins with ASA2/ASA3-like armadillo domains by DALI all-against-all search.** Proteins of known functions which are representative of cluster 1 are labelled and highlighted in blue, those representative of cluster 2 are labelled and highlighted in yellow. Proteins considered to belong to the same cluster as the representative proteins are framed in the same color. (A) Batch 1. (B) Batch 2. (C) Batch 3.
